# Supplementary material for: Are carbon emissions trading and green financial instruments synergistic? -Comprehensive quantitative research based on content analysis
Source: PLoS One. 2024 Mar 7;19(3):e0298601. doi: 10.1371/journal.pone.0298601 (PMC10919601; doi:10.1371/journal.pone.0298601)
Supplement: S1 Data — (ZIP) [file pone.0298601.s001.zip › Data/地级市Z_F工作报告.zip!/╣π╢1⁄2/╓Θ║ú╩╨/└┤╫╘╣1⁄2╓┌║┼í╛┬φ┐╦╩2╛▌═°í┐_2013.docx]

各位代表：

　　现在我向大会作政府工作报告，请代表们审议，并请市政协委员和其他列席人员提出意见。

　　一、2012年工作回顾

　　过去一年，是我市建设生态文明、推动科学发展取得新成就的重要一年。在市委的正确领导和市人大、市政协的监督支持下，市政府紧紧依靠全市人民，务实有为，扎实工作，较好完成了年度主要任务，开创了共建美丽城市、共享美好生活的新局面。

　　一年来，我们致力于多措并举稳增长，积极应对经济低迷、外需疲弱的困境，促进了经济平稳运行。预计完成地区生产总值1504.1亿元，比上年增长7%；人均生产总值9.5万元，约合1.5万美元，增长6.1%。公共财政预算收入162.6亿元，增长13.4%。城镇居民人均可支配收入32980元，增长14.8%。农村居民年人均纯收入13400元，增长13%。全市本外币存贷款余额分别为3449.7亿元和1920.3亿元，增长15.8%和17.2%。全市规模以上工业增加值644.8亿元，增长6.3%。完成全社会固定资产投资787.6亿元，增长23.6%，增速居全省前列。预计实际利用外资14.5亿美元、内资188亿元，分别增长8.2%和37.3%。社会消费品零售总额635.2亿元，增长12.7%。外贸进出口总额456.7亿美元，降幅由谷底的-14.2%收窄至-11.6%。

　　一年来，我们致力于加快交通基础设施建设，完善现代综合交通运输体系，确立了交通枢纽的地位。广珠铁路、广珠城际轨道全线贯通运营，圆了珠海百年“铁路梦”，标志着我市海陆空立体交通新格局已经形成。港珠澳大桥人工岛成型、主桥桥梁段和珠海连接线项目开工。高栏港高速、机场高速、珠海大道、明珠路等一批道路建、改工程竣工。珠海机场年旅客吞吐量突破200万人次。高栏港10万吨级主航道、3个10－15万吨级泊位等一批港口基础设施建成投产，全港吞吐能力达1.18亿吨。拱北口岸改扩建工程基本完工，启用后通关能力增加两倍多。

　　一年来，我们致力于构建“三高一特”现代产业体系，加快产业结构调整升级，产业整体素质有了新提高。海洋工程装备制造、通用航空等新兴产业发展壮大，预计先进制造业、高技术制造业增加值占规模以上工业增加值比重分别提升至45%和24%。高栏港经济区升级为国家级经济技术开发区，海洋工程装备制造基地成为省级战略性新兴产业基地，中海油系列项目加快建设，华润聚酯、珠江钢管一期和神华煤炭储运中心等项目建成投产，三一海洋重工产业园、中国北车珠海制造基地、银通新能源汽车等项目开工，瓦锡兰玉柴船用中速机、惠普智慧城市等项目落户。珠海航空产业园成为全国首个“国家通用航空固定运营基地发展示范区”，建成通用航空飞行服务站，中航通飞西锐飞机总装生产线投产，首架珠海制造公务机“领航150”样机下线。

　　现代商务、会展、旅游等产业加快发展，第三产业投资增长24%，预计现代服务业增加值比重提升至56%。第九届航展的规模、签约额均创历史新高，专业性、品牌效应提升。十字门会展商务组团建筑单体陆续封顶。长隆国际海洋度假区完成投资90亿元，海泉湾二期开工。保税区进口酒类交易中心和跨境工业区进口街建成运营。外经贸转型发展，一般贸易进出口占比提升至41.8%，委托设计和自主品牌混合生产方式出口所占比重上升10个百分点，加工贸易内销额增长9.2%。海洋经济和生态农业持续发展，海洋产业总产值增长13.6%，斗门生态农业园挂牌运作。民营经济稳步发展，民营工业增加值增长15%。“三清”工作深入推进，清理土地11932宗、政策1357项、项目1999个。节能减排成效明显，单位GDP能耗继续居全省第三低位。创新驱动能力增强，全市科技支出占财政支出的4.2%，发明专利申请量和授权量增长59％和70%。高层次、高技能和创新型人才队伍壮大，横琴获批“全国人才管理改革试验区”，丽珠集团蛋白质药物研究开发及产业化团队入选广东省第三批引进创新科研团队，一批高技能人才入户珠海。

　　一年来，我们致力于推动横琴加速崛起，打造现代服务经济发展集聚区和深化粤港澳合作新载体，新区开发取得了重大进展。环岛东路等多条岛内主干道建成通车，横琴二桥动工，广珠城轨延长线项目、金海大桥加紧推进，水电气等市政配套设施建成使用，横琴新家园首期工程完工。《横琴产业发展指导目录》通过国家发改委初审，总投资超过1750亿元的41个重点高端产业项目加快建设。横琴金融服务产业基地一期投入使用，引进金融类机构和企业100多家。珠港澳更紧密合作开发横琴工作取得实效，横琴岛澳门大学新校区主体工程基本建成，粤澳合作中医药科技产业园等项目扎实推进。《珠海经济特区横琴新区条例》、《珠海经济特区横琴新区商事登记管理办法》、《海关总署关于支持横琴新区开放开发的意见》、省政府《关于加快横琴开发建设的若干意见》等法规政策陆续实施，产业优惠目录和文化创意先行先试政策等正抓紧报批。

　　一年来，我们致力于优化提升宜居环境，建设美丽城市，城乡区域发展更趋协调。大力创建全国文明城市和全国生态文明示范市，新一轮全国城市文明程度指数测评成绩显著提高，通过国家环保模范城复核，连续十三年获评省环境保护责任考核优秀，香洲区建成“国家生态区”。新建（改造）各类公园29个、生态景观林带57公里、绿道90公里，治理闲置和违法用地6784亩，拆除违法建筑33万平方米。绿色交通加快发展，新投放新能源公交车400辆，建成公共自行车租赁系统首期195个租赁点，启动中心城区1号线有轨电车试点项目。智慧城市建设取得成效，互联网普及率、家庭宽带普及率和无线宽带网络覆盖率均超过78%，居全省前列。城市组团综合功能增强，市政府投资的40%投向西部地区等新开发区域，落实了西部中心城区首期项目投资主体，实施东部主城区拆建类城市更新项目44个。幸福村居创建全面启动，斗门区北部四小联围海堤加固达标工程等农村基础设施建设取得新进展。珠中江区域合作33个项目深入实施，“双转移”、扶贫开发“双到”、对口凉山州东西扶贫协作等年度帮扶任务全面完成。

　　一年来，我们致力于增进民生福祉，全面加强社会建设，共享美好生活取得了新成果。全市财政九项民生支出122.2亿元，增长34.7%，占公共财政预算支出的59.5%。就业形势稳定，城镇登记失业率为2.3%，城镇新增就业人数43815人。社会保险工作加强，全市参保总人次达484万，全市企业退休人员月平均养老金提高至2291元、增幅为21%，居民医保住院费用基金平均支付比例达到75%、支付限额提高到20万元，保障水平居全国前列。社会养老服务体系加快建设，全市居家养老服务对象发展至600余人，机构养老床位总数增加到2200张。低收入群众生活保障加强，城乡居民最低生活保障标准调至400元和350元，增长25%和14.3%。保障性住房建设有新进展，新开工建设保障性住房、棚户区改造住房近3000套（户），竣工3300套（户），发放住房补贴316.9万元。新增积分入户的优秀异地务工人员2800名。“校安工程”三年规划加固任务全面完成，建成文园中学新校区和11所镇中心幼儿园，新增学位8000多个，14840名进城务工人员随迁子女新入读义务教育公办学校。医疗基础设施加快完善，推进医疗就诊“一卡通”项目，探索第三方评价、医疗责任险等缓解医患矛盾新机制，医疗卫生服务水平有新提高。投入1.5亿元加快推进“一院三馆”项目建设和镇（街）、村（居）文化设施全覆盖工程，定时免费开放市体育中心场馆和圆明新园等公共文体设施。人口均衡型社会示范市加快建设，人口计生工作保持全国领先水平，人口自然增长率为9.2‰。国防和民兵预备役建设加强，荣获全国双拥模范城“七连冠”。平安珠海建设深入推进，安全生产、食品药品和农产品安全监管及“三打两建”、应急管理、防灾减灾、外事侨务、民族宗教、妇女儿童等工作扎实有效，社会保持和谐稳定，各项社会事业全面发展。

　　一年来，我们致力于全面深化改革和优化政府服务，创新科学发展体制机制，增强了经济社会发展的动力和活力。国有经济布局和结构优化，81%的经营性资产实现了产权多元化，市管企业通过发行企业债等方式融资294.6亿元。珠海农信联社改制为珠海农商银行，营业税改征增值税试点启动，中小企业区域集优债发行等金融创新工作加快落实。省下达的医疗卫生体制改革目标任务基本完成。社会组织登记管理改革深化，新登记社会组织132家，增加11.5%。社会组织承接政府转移职能50项，确定政府购买服务五大类283项。社会工作专业人才队伍建设加强，新增持证社工166人。社区“六个一”服务平台建设试点工作稳步推进。“两建”综合试点工作加快实施，出台《珠海经济特区商事登记条例》，率先在全国进行商事登记制度改革，率先在全省开通“12345”市民服务热线。启动第五轮行政审批制度改革，进一步简政放权。落实政府部门白皮书制度，2012年《政府工作报告》各项任务完成率达98.3%。全面开展国家级行政服务标准化试点工作，建立124个镇（街）便民服务中心和村（居）代办点。发展电子政务，网上办事大厅加快建设，市、区98个部门、1267个事项完成网上注册。坚持科学、民主、依法决策，自觉接受人大、政协和社会监督，提请市人大常委会审议地方性法规5件，制定修改政府规章9件。办理人大议案3件、代表建议181件、政协提案352件，解决了一批人民群众关注的问题。加强反腐倡廉和公务员队伍建设，机关作风和行政效能进一步改善。

　　各位代表！回顾过去一年，我们按照“科学发展走新路、‘十二五’崛起看珠海”的总要求，以全球视野和战略思维多方求解经济转型、环境宜居、社会治理、文化建设、法治保障等重大现实课题，进一步明确了建设生态文明、实现科学发展的思路和举措。面对年初开局经济下行的不利形势，我们把稳增长放在更加重要的位置，及时采取了鼓励扩大投资、发展总部经济、扶持民营和中小微企业、稳外贸促增长等一系列积极举措，实现了全年经济“首季筑底、逐季回升”。我们坚定不移地推进产业结构战略性调整，进一步完善产业发展规划和配套政策，加快横琴、高栏、高新区等重大平台和产业基地建设，引导产业高端发展，推动形成新的经济增长极。我们紧紧抓住面临的战略性机遇，以基础设施、市政建设为重点，狠抓力促投资持续较快增长这个关键，破解制约珠海发展的瓶颈，有力支撑了经济增长、产业转型和城市升级。我们以创建全国生态文明示范市为战略抓手，实施了一批环保基础设施、城市绿化美化等绿色生态工程，提高了城市宜居水平。我们把保障和改善民生作为政府工作的主要任务，积极推进社会保障全覆盖和基本公共服务均等化，办成了一批让人民群众得实惠的好事实事。一年来，政府各项工作的顺利开展与取得的成效，得益于全市人民的团结奋斗和社会各界的关心支持。在此，我代表市政府，向广大人民群众和社会各界人士表示衷心的感谢！

　　毋庸讳言，我市经济社会发展还存在不少矛盾和问题：一是在外部经济增长下行压力持续不减和经济结构性矛盾依然突出的双重影响之下，经济增长未能实现年初预定目标，经济增长仍然没有摆脱过度依赖以加工贸易为主的外贸出口和房地产投资的局面。新产业、新项目大多处在建设期，民营经济不发达、规模小，两者对增长贡献还不显著，调结构、促增长统筹平衡的压力增大。二是自主创新体系建设面临资源分散、人才缺乏等问题，自主创新能力有待进一步增强。三是城市宜居水平与市民期待有一定差距，一些市政基础设施尚需完善，交通出行便利化程度尚需加强，城市管理精细化水平尚待提高，城乡环境有待优化提升。四是公共服务供给和需求还不平衡，基本公共服务均等化还存在较大差距，城乡发展不平衡的问题仍然存在。五是体制机制改革更显紧迫，政府的管理服务亟待创新，法治化国际化营商环境仍需优化。对此，我们将高度重视，采取切实措施，逐步解决。

　　二、2013年重点任务

　　党的十八大把“生态文明”纳入“五位一体”总布局，强调要把生态文明建设融入经济、政治、文化、社会建设各方面和全过程。去年12月，习近平总书记视察珠海时殷切寄语我们，坚持生态文明新特区、科学发展示范市的定位，沿着这条道路扎实推进、大胆探索，展现出的将是一个具有独特优势、前景光明的新珠海。我们肩负的使命光荣而艰巨，要以时不我待的危机感、只争朝夕的紧迫感，坚毅自信、心无旁骛、励精图治、奋发图强，朝着建设生态文明、推动科学发展的正确方向加速前行。我们要进一步强化创新驱动，加快转变经济发展方式，加快提升经济参与全球中高端竞争的综合实力。我们要进一步深化改革开放，着重营造法治化国际化营商环境，加快使法治成为经济社会运行的基本保障。我们要加快健全和完善党委领导、政府负责、社会协同、公众参与、法治保障的社会管理格局，构建政府、社会、公众共建共治共享的社会管理新机制，加快形成与市场经济要求相适应的社会治理模式。我们要着力创建全国文明城市和全国生态文明示范市，加强社会主义核心价值体系建设，加快建立与科学发展要求相适应的主流价值观，加快打造与欧美先进国家相媲美的宜居环境。我们要加快推进政府改革，处理好政府、市场与社会三者之间的关系，加快建设与社会管理创新要求相适应的“管理的小政府、服务的强政府”。

　　综合判断今年的发展环境，世界经济低速增长态势仍将延续，我市产业发展正处在转型升级的爬坡过坎阶段，一批引领未来、支撑发展的重大项目建成达产尚需时日，新经济增长点还在形成，政府投资的资金筹措也越来越难。但国内发展基本面长期趋好，中央宏观调控政策保持积极而稳健态势，近年来我市大力发展生态文明、奋力建设珠江口西岸核心城市，开发横琴新区、建设交通枢纽、打造产业基地等迸发的发展活力和动力日益强盛。总体上，我市发展具备难得的机遇和有利条件，扩大内需、加快投资和调整结构、转型升级都大有作为。

　　我们要深入贯彻落实党的十八大、中央经济工作会议和省委十一届二次全会精神，坚持建设生态文明新特区、科学发展示范市的目标定位不动摇，实施“蓝色珠海、科学崛起”发展战略，坚持稳中求进，促进加快发展，在推动发展中加快转变经济发展方式，加大经济结构战略性调整力度，强化创新驱动发展，着力保障和改善民生，增强经济发展的内生动力和活力，努力实现更好质量、更高效益的稳定增长和可持续的全面发展。

　　今年经济社会发展的主要预期目标是：实现地区生产总值增长9%左右；规模以上工业增加值增长10%；全社会固定资产投资增长15%；社会消费品零售额增长13%；外贸出口总额增长11%；地方公共财政预算收入增长12%；城镇居民年人均可支配收入和农村居民年人均纯收入分别增长10%、11%；城镇登记失业率控制在3.2%以内；户籍人口自然增长率控制在9.8‰以内；完成省下达的节能减排目标任务。

　　围绕实现上述目标，要重点抓好以下工作：

　　（一）促进经济持续健康发展。

　　保持投资合理较快增长。今年政府投资计划安排108.9亿元，其中 67亿元用于建设交通、生态等项目。继续推进“十大重点工程”建设。提高产业投资比重。鼓励扩大民间投资。落实面向民间投资的8个重点项目，研究推出一批新项目。

　　积极扩大消费需求。实施收入倍增计划，落实收入分配改革和结构性减税政策，稳步提高最低工资标准。精心办好中国国际马戏节等重大活动，促进文化旅游消费。培育电子商务平台和网商，支持企业应用和发展电子商务，发展网购、团购等新兴消费业态。拓展家政等生活服务消费领域。做好农超对接、“万村千乡”、农贸市场改造等工作，繁荣农村消费市场。

　　促进外贸转型发展。支持企业稳住传统市场、积极开拓新兴市场。推动加工贸易转型升级，发展服务贸易和国际中转、采购、配送和转口贸易。支持企业开展跨国经营、跨国并购、建立生产基地和营销网络。鼓励扩大能源、重要原材料、先进适用技术及设备、关键零部件等重点商品进口。继续支持保税区、跨境工业区建设进口商品基地。完善外贸协调机制，加强通关便利化工作。

　　加强经济运行调节。及时监测分析经济运行情况。抓好煤电油气运的运行保障和重要农副产品供给。加强市场监管和治理整顿，保持市场和物价稳定。继续挂点帮扶重点企业，为企业排忧解难。

　　（二）加快产业结构调整升级。

　　全面提升自主创新能力。全面落实科技创新促进条例、所得税优惠税率、企业研究开发费税前扣除等政策，实现R&D投入占比达2.1%。更加注重发挥企业家才能，培育企业家精神和企业家队伍，强化企业创新主体地位。深化与高校的战略合作，提升大学园区发展水平。增强研发公共服务平台功能，推进国家海洋工程及船舶材料检验中心等技术创新和服务平台建设，新增一批企业技术中心和工程中心。推进质量强市建设，加强知识产权、技术标准和名牌带动工作，形成一批具有自主知识产权的专利技术和产品，制定一批先进标准，提升产业技术创新和应用水平。健全科技投融资体制，探索推广“三资融合”建设模式，发展创业投资和股权投资，鼓励银行加大对科技型中小企业的信贷支持，推动科技企业上市融资。落实中长期人才发展规划纲要和人才政策，培养集聚创新创业人才。

　　加快现代产业体系建设。完善“三高一特”产业专项规划和配套政策。继续培育53个战略性新兴产业重点项目，推进航空、软件与集成电路设计、海洋工程装备制造、新能源客车4个省级战略性新兴产业基地建设。发挥海港、空港、铁路的综合效用，申报高栏港综合保税区，建设港口和广珠铁路物流园，大力发展现代物流业。扩大横琴政策效应，大力发展实业导向型和政策导向型区域总部经济。完善金融综合服务体系，推动金融创新，设立珠海创业投资引导基金，培育大型金融控股企业，壮大现代金融产业。编制实施会展产业发展规划，筹建行业主管机构和国有会展集团，加强与国内外专业会展机构的合作，发挥中国航展的品牌效应，培育一批具有较大影响力的专业展会。规划建设若干游艇对外开放码头，发展一批游艇会所，促进游艇消费、服务和运营，推动游艇研发和制造。推动横琴长隆、海泉湾二期、东澳岛玲玎海岸等重大项目建设，发展温泉、海岛、高尔夫等特色旅游业，加快打造国际商务休闲旅游度假区。推动国际耗材采购中心和联想赛纳打印机研发制造基地建设，促进打印耗材产业转型发展。

　　服务大项目培育大企业。深入推进“三清”工作，提高发展承载能力。规划建设生物医药产业专区，完善金湾航空新城、富山工业新城等基础配套。开工建设碧辟化工PTA三期、华润热电等项目。协调推进中国北车珠海制造基地、瓦锡兰玉柴船用中速机、金山南方总部等项目。加快建成十字门中央商务区一期、中海油系列项目、三一海洋重工产业园、珠江钢管二期等项目。促进中航通飞珠海制造基地等新竣工项目投产达效，加快中国民用航空飞行校验中心南方基地建设。坚持招强引优，突出总部招商，实现实际利用外资增长不低于6%。

　　着力发展“蓝色经济”。创建国家级海洋生态文明示范区，实现海洋产业总产值增长13%。深化海洋经济发展规划，修编《万山区总体规划》等专项规划。培育海洋可再生能源等新型海洋技术产业，创建珠海（万山）海洋科技示范园。加快海港枢纽、海洋防灾减灾、海岛能源保障工程建设，完善涉海基础设施。有序开发无居民海岛。加强海洋生态保护，划定禁止网捕区域，扩大海岛生态修复试点成果。

　　扶持民营和中小微企业发展。进一步完善服务体系，加强民营企业在用地、人才、融资、市场开拓等方面的政策支持和管理服务。从今年起，市级财政预算新增1亿元用于发展民营经济。放宽市场准入，鼓励民间资本参与基础设施和公用事业项目投资，支持小微型企业参与政府采购。积极发展小型金融机构，完善创业投资扶持机制，拓宽民营和中小微企业融资渠道。组织上市公司和中小微民营企业资金、项目对接活动，实现大中小企业互利共赢、抱团发展。

　　（三）推进交通基础设施现代化。

　　加快洪湾至高栏港高速、香海高速、现代有轨电车一号线、国道G105快速干道等项目前期工作，力争年内开工。开工建设情侣路南段改造、金湾立交等项目。推进港珠澳大桥珠海连接线、太澳高速珠海大道至横琴段、珠海大道金湾互通至高栏港段改造、省道272线斗门段、金琴高速、横琴二桥、西沥大桥等项目建设。建成高栏港疏港铁路专用线一期主体工程、港珠澳大桥珠澳口岸人工岛填海工程等项目。加大口岸资源整合力度，完善拱北口岸改扩建工程，加快港珠澳大桥珠海口岸规划建设，完成珠澳跨境工业区专用口岸扩大功能项目研究工作。推进现代化国际港口、信息化数字港口建设。建设高栏港10万吨级集装箱码头泊位、10万吨级秦发煤码头泊位和15万吨级主航道扩建等项目，实现港口货物吞吐量过亿吨。推进九洲港货运码头搬迁至洪湾港工作。启动珠海机场第二跑道建设前期工作，协调推进珠海机场对外开放，建设珠海通用机场。

　　（四）加快横琴新区开发开放。

　　落实创新政策。贯彻已颁布的各项法规政策，推动出台横琴产业发展优惠目录、海关监管、税收征管等政策。制定实施创建全国人才管理改革试验区具体政策，推动诚信体系建章立制工作，引进国内著名仲裁机构。做好横琴口岸创新通关模式的细化和实施工作。

　　完善基础配套。全面启动综合开发项目建设。开工建设广珠城轨经横琴至珠海机场段。推进金海大桥等陆岛连接项目。建设横琴口岸及综合交通枢纽等基础设施，新建直升飞机起降点、游艇码头和临时客运码头，开辟海上客运线，打造立体交通网络。建立绿色建筑评价机制，规划建设岛内绿色交通体系。

　　发展高端产业。促成长隆国际海洋度假区一期、多联供燃气能源站等项目竣工，推进横琴总部大厦、客商汇南方总部、香洲埠文化院街、丽新星艺文创天地等项目。深化金融创新，争取筹建各类新型金融机构和交易市场，推动外资金融机构设立分支机构，吸引国内各类金融机构发展跨境人民币业务，开展资本项目可兑换的先行试验。加快粤澳合作中医药科技产业园建设和招商工作，推动粤港澳文化创意产业园规划建设。

　　（五）提高城乡宜居水平。

　　优化城市环境。完善城市规划体系，推进“五规融合”。加强与专家团队的战略合作，完成城市总体规划修改和城市概念性空间发展规划，加快全市建设用地控规全覆盖工作。研究利用港珠澳大桥牵引效应，增强城市功能。改造情侣路沿线海湾及前山河、黄杨河“一河两岸”等重点片区。推进香洲渔港整体搬迁工作。完成海滨泳场“动感舞台”改造工程。打造“九洲动感活力轴”，推进市民文化广场、九洲商贸中心等城市更新项目，新开工建设2-3个城市综合体。启动拱北口岸地区综合改造开发，实施沥溪、南溪、福溪片区人居环境改善工程。推进金鼎、上冲门户地区以及港湾大道、梅华路、粤海路、石花路等改造，争取部分项目年内开工。推进西部城区开发，开发首期开发区域核心片区，动工建设航空新城片区和白藤片区。启动平沙新城和金湾西湖景观提升工程建设。推进广佛江珠城际轨道及湖心路口换乘枢纽等项目。

　　加快智慧城市建设。完成智慧城市规划编制工作，构筑智慧城市建设框架。年内建成网上办事大厅。建设市民网页支撑平台。制定智能交通规划，上半年完成交通信息综合服务平台项目建设。扩大口岸电子验放系统应用，建设物流电子口岸信息共享平台。加大信息基础设施建设力度，推进光纤入小区、入户，提升高速宽带网络普及率。启动城市云计算中心建设，在软件、集成电路设计、新一代宽带移动通信、物联网等领域寻求新突破。

　　提高城市精细化管理水平。推动制订城市管理条例。理顺城市管理体制，将城市管理重心下移、事权下放。开展“城市管理提升年”活动，加强公共环境卫生、农贸市场和户外广告整治。推进“数字城管”二期建设，完善城市管理信息体系。促进绿色出行，优先发展公共交通。优化公交线网密度和站点覆盖率，提高公共交通出行分担比率。加大绿色能源和新能源汽车投放力度，完成500辆绿色公交车转换工作。完善人车分流交通运输系统，建设一批人行过街设施。优化公共自行车租赁系统运营管理，新建200个租赁点、投放5000辆自行车。继续抓好绿道网建设和运营，新建省立绿道60公里。

　　共建优质生活圈。推动珠中江交通基础设施一体化，打通造贝工人新村路、金鸡路等城际断头路，增大市际客运专线和公交班车的运力。推动珠中江供水同网工程建设。落实医学检验影像检查结果互认及病历“一本通”制度。探索港澳居民和医生到珠海就医、执业等医疗卫生合作新模式，逐步扩大开放珠海医疗服务市场。广泛开展珠中江澳群众性文体旅游活动。开展珠澳跨境交通发展研究。继续抓好市外结对帮扶工作。

　　（六）推动城乡一体化发展。

　　加快发展现代农业。完善斗门生态农业园开发建设政策、土地、资金扶持机制，加快打造全国一流农业科技示范园。支持台湾农民创业园加快发展。推动农渔民转业转产，依托特色优势农产品促进深加工产业发展壮大。加强高标准基本农田和农村水利建设。加快完成乾务赤坎大联围、小林联围及斗门区北部四小联围海堤加固达标工程。

　　增强农村发展活力。稳妥推进城镇化，加强中心镇、专业镇、特色镇建设，发展镇域经济，促进镇村互动发展。加强农村规划建设、农村产权制度改革、土地流转等方面的立法。推进创建幸福村居“六大工程”和农村农业体制机制改革创新工作。完善市、区、镇（街）三级工作机构，落实结对帮扶和监督考核工作。深化村居产业发展、公共服务、基础设施、社会治理等问题研究，指导村居规划建设。重点扶持10个先进示范性村居，形成辐射带动效应。继续推进自然村通水泥路工程，基本完成46座危桥改建任务，推进低标准县道升级改造工作。

　　持续改善农民生活。完善农村集体土地使用管理机制，出台集体经济组织成员资格界定及利益分配的指导性政策，继续探索推广村（农村股份合作公司）集体留用地开发配套园区试点工作。落实基本农田保护区、生态保护区、水源保护区的扶持激励机制，加大市内欠发达村（农村股份合作公司）的扶持力度。推进历史文化名城、名镇、名村建设，综合保护和开发利用唐家湾、斗门古镇及北山、会同、南门、网山、排山古村等历史文化古迹。加大农村学校教师培训力度，新建10所镇中心幼儿园。推进西部地区医疗机构建设，提升海岛医疗卫生应急救治能力。改善西部农村地区公共体育设施条件，启动镇一级公共体育健身广场建设。进一步完善政策，切实解决好农民建房住房问题。

　　（七）大力建设生态文明。

　　加大生态环境保护力度。建设生态环境监测监控系统。强化机动车污染防治。继续开展裸露山体复绿工作，加强自然保护区和湿地资源保护。完成凤凰山森林公园建设一期工程，新建生态景观林带85公里，绿化美化一批市政道路。加快推进东部城区污水处理厂新改扩建项目社会化投融资建设和特许经营。实施城镇内涝排渍改造工程。加快西区污水处理厂和管网建设，实施河涌排洪渠截污综合整治和雨污分流规划改造。推进西部垃圾无害化处理中心、市生活垃圾处理环保工厂建设。实施生活垃圾分类和无害化、资源化处理利用。启动核应急体系建设，维护环境安全。加快创建全国生态文明示范市，推动金湾、斗门建成省级和国家级生态区，井岸、白蕉、乾务、莲洲、万山、担杆六个镇建成国家级生态镇，各级生态示范区（镇、村）覆盖率达80%，“绿色学校”、“绿色社区”增长比率达10%。

　　促进资源节约集约利用。健全节能减排统计监测和考核评价制度，严格落实节能减排目标责任制，全面完成省下达的目标任务。抓好工业、建筑、交通运输和公共机构等领域的节能，加强对污染减排、重污染行业以及中小型污染企业的执法监管。全面推进低碳试点城市建设，加快建设园区集中供热项目，推进高栏港建设循环经济试点园区，推动横琴低碳岛建设，支持高新区创建国家级生态工业示范园区。落实最严格的土地管理制度，优化国土空间开发格局，合理控制土地开发强度，深入推进“两违”整治，加强国土监察执法，合理、有序规划利用城市地下空间，提高土地利用效率和投入产出水平。

　　完善生态文明建设制度。办好生态文明珠海研究院，开展生态文明建设体制、机制和法制研究。建立健全国土空间开发保护、饮用水源保护、耕地保护和环境保护制度。积极开展环保自觉公益行动，倡导健康文明、低碳环保的生活方式和消费模式。

　　（八）持续推进文化强市建设。

　　完善公共文化服务体系。继续推进珠海歌剧院和博物馆、城市规划展览馆建设。打造圆明新园文化艺术中心，年内完成园内及文化馆改造工程。推进十字门中央商务区音乐厅、香洲文化及体育中心等文化工程。建设留学生历史博物馆。健全市、区、镇（街）、村（居）四级公共文化设施网络，全面实现广播电视户户通，推动一批镇（街）文化站创建省特级文化站。启动市综合性科普与公共安全教育场馆建设，加快建设国家数字图书馆和公共电子阅览系统。

　　提升城市文明程度和文化活力。推进全国文明城市创建工作，实施“德行珠海”公民道德建设行动计划，广泛开展“明德讲堂”活动，加强未成年人思想道德建设。加强文化产品和文化服务知识产权保护力度，实施文艺精品工程，完善文艺精品创作激励机制，扶持代表地方特色的文艺作品。调动社会力量参与支持文化建设，广泛开展群众性文化活动。编制文化人才引进需求目录，引进高端文化人才及团队，造就一批有影响力的文化代表人物。

　　加快发展文化创意产业。研究制定文化创意产业扶持政策，重点发展数字内容产业、文化旅游产业、创意设计产业、文化产品制造业以及文化产品与服务出口。规划建设横琴“粤港澳文化创意产业园区”。加快珠海南方影视文化产业基地、南屏V12文化创意产业园等龙头项目建设。

　　（九）切实保障和改善民生。

　　提高基本公共服务均等化水平。加快创建广东省推进教育现代化先进市。加快实施“强师工程”，深化高中阶段学校招生制度改革。推进小区配套幼儿园建设及移交工作，合理提高公办幼儿园比例，规范化比例达70%以上。推进金湾一中、市理工职业技术学校斗门校区二期、市技工学校新校区一期、市特殊教育学校康复训练大楼、斗门区特殊教育学校、联合国际学院新校区、格力工程技术学院等项目建设。推动市妇幼保健院二期、市慢性病防治中心（市精神卫生中心）、市紧急医疗救援中心、市第二中医院等建设项目前期工作，启动横琴新区综合医院建设，继续改造一批薄弱卫生院。完善医疗就诊“一卡通”项目，打造全省卫生信息化建设工作亮点。推进公立医院改革，实施医师多点执业、公立民营联建协管等制度，提升整体医疗服务能力。

　　健全社会保障体系。建立健全以公租房为主的新型住房保障体系。新开工建设保障性住房2000套，竣工3264套。加强准入、后续和退出管理，促进保障性住房分配公开、公平、公正。落实调控政策，促进房地产市场平稳健康发展。实施更加积极的就业政策，促进高质量就业，城镇新增就业人数4万人，实现就业困难人员就业2000人。全面推进企业工资集体协商工作，实现已建工会企业的建制率达85%以上。加强劳动保障监察，指导企业规范劳动用工管理，创建和谐劳动关系。大幅提高新农保和城居保政府补贴标准，基础养老金由每人每月165元提高至330元，新农保老年津贴由原每人每月165元提高至200元。引入市场机制，建立补充医疗保险制度，进一步提高全市医疗保障水平。修订生育保险制度，进一步保障职工生育权益。推进社会养老服务体系建设“五个一”工程。推动人口均衡型社会示范市建设，继续实施优生惠民工程，完善人口信息化网络，改善流动人口服务管理。规范物业管理收费，加强小区物业管理工作。扎实推进平安珠海创建工作。进一步落实安全生产“一岗双责”，率先创建全国安全发展示范市。加强治安技防，实现“三联”村居创建率和“技防村居”覆盖率超过30%。贯彻《珠海市见义勇为人员奖励和保障条例》，弘扬社会正气。

　　加强社会管理创新。建立行政事务村（居）准入机制，加快村（居）民自治和城乡社区建设，50%行政村建立村务监督委员会制度，30%行政村达到省村务公开和民主管理示范创建标准。注重发挥工会、共青团、妇联在构建枢纽型社会组织中的重要作用，引入知名机构管理运营社会组织孵化基地，深化社会组织登记管理制度改革，培育和扶持社会组织健康发展。建立第二批具备承接政府职能转移和购买服务资质的社会组织目录。加强社会组织规范化管理，拓宽社会组织依法参政议政渠道。开展购买社工服务试点工作。建立健全社会工作专业人才激励保障政策，推行“社工+志愿者”服务模式，加强与港澳社会服务机构的合作，扩大服务覆盖面。加快建设志愿服务阵地，开展“三关爱”志愿服务活动。

　　（十）优化提升营商环境。

　　纵深推进重点领域改革。理顺市、区、镇三级管理体制，进一步简政放权。推进第五轮行政审批制度改革，行政审批事项减少40%、行政审批总时限压缩50%以上。推进竞争性国企引进战略合作伙伴、公益性国企特许经营改革，全面深化董事会建设工作，加强投资管理，完善收入分配制度。深化财政体制改革，完善促进基本公共服务均等化的公共财政体系。

　　抓好“两建”工作。落实社会信用体系、市场监管体系建设实施方案。推进信用法制体系、行业和部门信用、公共联合征信系统及政务诚信建设，加强信用服务市场的培育和监管，建设社会信用信息和市场监管信息平台，促进信用信息互通共享。加快完善“12345”市民服务热线运行保障措施。创新食品安全监管机制，提高食品安全保障水平。

　　全面加强政府服务。坚持依法行政，提高行政权力运行的制度化、法治化水平。扎实推进“五好”领导班子建设，不断增强推动科学发展的能力。加快发展电子政务，优化政务服务。完善政务公开和各领域办事公开制度，促进行政权力运行公开化，有效接受监督。坚持问政于民、问计于民、问需于民，改进工作作风，密切联系群众。严格控制“三公”消费和一般性开支，降低行政成本。加强行政监察、审计监督等工作，强化廉政风险防控，严查腐败案件，推进廉洁城市建设。

　　各位代表！美丽城市是我们的共同家园，美好生活是我们的共同向往。建设美丽城市为了人民，美好生活属于人民。广大人民群众始终是美丽城市和美好生活的创造者和享有者。让我们在市委的坚强领导下，为实现共建美丽城市、共享美好生活的城市理想，加快建设生态文明新特区、科学发展示范市而努力奋斗！
